# Comparative Evaluation of Two Glass Polyalkenoate Cements: An In Vivo Pilot Study Using a Sheep Model

**DOI:** 10.3390/jfb12030044

**Published:** 2021-08-05

**Authors:** Leyla Hasandoost, Daniella Marx, Paul Zalzal, Oleg Safir, Mark Hurtig, Cina Mehrvar, Stephen D. Waldman, Marcello Papini, Mark R. Towler

**Affiliations:** 1 Faculty of Engineering and Architectural Science, Biomedical Engineering Program, Ryerson University, Toronto, ON M5B 2K3, Canada; leyla.hasandoost@ryerson.ca (L.H.); dmarx@ryerson.ca (D.M.); swaldman@ryerson.ca (S.D.W.); mpapini@ryerson.ca (M.P.); 2 Li Ka Shing Knowledge Institute, St. Michael’s Hospital, Toronto, ON M5B 1W8, Canada; 3 Faculty of Medicine, Department of Surgery, McMaster University, Hamilton, ON L8S 4L8, Canada; paulzalzal@gmail.com; 4 Oakville Trafalgar Memorial Hospital, Oakville, ON L6J 3L7, Canada; 5 Division of Orthopedic Surgery, Mount Sinai Hospital, 600 University Ave, Toronto, ON M5G 1X5, Canada; Oleg.Safir@sinaihealth.ca; 6 Ontario Veterinary College, University of Guelph, 50 Stone Rd E, Guelph, ON N1G 2W1, Canada; mark.hurtig@gmail.com; 7 Department of Mechanical & Industrial Engineering, Ryerson University, Toronto, ON M5B 2K3, Canada; cmehrvar@ryerson.ca; 8 Department of Chemical Engineering, Ryerson University, Toronto, ON M5B 2K3, Canada

**Keywords:** glass polyalkenoate cement, revision total knee arthroplasty, in vivo, sheep, bone cement

## Abstract

Poly(methyl methacrylate) (PMMA) is used to manage bone loss in revision total knee arthroplasty (rTKA). However, the application of PMMA has been associated with complications such as volumetric shrinkage, necrosis, wear debris, and loosening. Glass polyalkenoate cements (GPCs) have potential bone cementation applications. Unlike PMMA, GPC does not undergo volumetric shrinkage, adheres chemically to bone, and does not undergo an exothermic setting reaction. In this study, two different compositions of GPCs (GPCA and GPCB), based on the patented glass system SiO_2_-CaO-SrO-P_2_O_5_-Ta_2_O_5_, were investigated. Working and setting times, pH, ion release, compressive strength, and cytotoxicity of each composition were assessed, and based on the results of these tests, three sets of samples from GPCA were implanted into the distal femur and proximal tibia of three sheep (alongside PMMA as control). Clinical CT scans and micro-CT images obtained at 0, 6, and 12 weeks revealed the varied radiological responses of sheep bone to GPCA. One GPCA sample (implanted in the sheep for 12 weeks) was characterized with no bone resorption. Furthermore, a continuous bone–cement interface was observed in the CT images of this sample. The other implanted GPCA showed a thin radiolucent border at six weeks, indicating some bone resorption occurred. The third sample showed extensive bone resorption at both six and 12 weeks. Possible speculative factors that might be involved in the varied response can be: excessive Zn^2+^ ion release, low pH, mixing variability, and difficulty in inserting the samples into different parts of the sheep bone.

## 1. Introduction

Revision total knee arthroplasty (rTKA) rates have dramatically increased worldwide in recent years. The total annual number of rTKA surgeries is forecast to rapidly increase by 182% in 2014 to 2030 in the United States (U.S.) [1]. In Canada, more than 75,000 rTKA surgeries were performed between 2018 and 2019, reflecting a 22.5% increase compared to five years earlier [2]. While rTKA surgeries comprise a relatively small percentage of all joint replacements (and approximately 7% of all knee replacements [2]), revision surgeries are more complex than primary surgical procedures, resulting in higher inpatient costs (nearly double that of the primary total knee arthroplasty (TKA)), decreased function of the knee, and longer patient recovery time [2]. To manage bone loss in rKTA, surgical implants and techniques such as augments, sleeves, cones, bone grafts, and cementation can be used, depending on the bone defect size, bone quality, and patient’s age [3,4,5,6]. The following table reviews each treatment option currently available in rTKA.

As can be seen in Table 1, PMMA bone cement is one of the void fillers currently used in rTKA. However, the application of PMMA is limited to bone defects, which have a depth of less than 5 mm and cover less than 50% of the area of implant/osseous interface [7,8]. Moreover, the use of PMMA results in thermal necrosis (due to heat generation during the polymerization process), aseptic loosening [9], wear debris [10,11], and volumetric shrinkage [12]. The inability to bond chemically to the bone surface results in failure at the bone–PMMA interface and loosening [9]. Due to these significant complications posed by using PMMA following rTKA, there has been a demand for a novel bone cement for managing bone loss.

Glass polyalkenoate cement (GPC) was developed in the early 1970s. The cement sets by an acid-base reaction between polyalkenoate acid and *fluoro-alumino-silicate* glass [13,14,15,16]. GPCs have been used in dentistry because they present advantages such as single-step adhesion to enamel and dentine, hydrophilicity, biocompatibility, and dimensional stability [13,17]. However, the use of GPCs in orthopedic applications has been limited to the inner ear and for craniofacial implants [18,19]. Commercial GPCs (e.g., Fuji IX GP (GC Europe NV, Leuven, Belgium, and Ketac Cem Easymix (3M ESPE, St. Paul, MN, USA)) contain aluminum (Al) [20,21,22], which can have a deleterious effect on bone mineralization [23,24]. Therefore, attempts have been made to change the chemistry of GPCs and formulate a GPC without aluminum [14,25]. Towler et al. [26] developed and patented an Al-free glass (mol%: SiO_2_: 0.48, ZnO: 0.36, CaO: 0.12, SrO: 0.04) (US 7,981,972)) based on the replacement of the Al component by zinc (Zn), an essential element for proper cellular and immune function and effective wound healing [14,27]. Moreover, Sr was incorporated as it facilitates bone growth and mineralization [28]. Subsequently, several Sr containing GPCs were formulated with adjustable working times from 50 to 120 s. In 2016, Alhalawani et al. investigated the effect of the substitution of ZnO by Ta_2_O_5_ [29]. Tantalum (Ta) is a transitional metal that acts as a glass-former due to the basic unit of tetrahedra chains linked together through corners. The results from Alhalawani et al. showed that the addition of Ta_2_O_5_ causes a change in the GPC network, which leads to an increase in network connectivity and glass thermal stability [29]. Based on the positive results from this study, tantalum-containing ionomeric glass was fabricated (US 10,815,144) [30] and the resultant GPCs were investigated in vitro [31]. However, to date, no in vivo study has been performed to assess the biocompatibility of tantalum-containing GPC.

In orthopedics research, the state-of-the-art is such that in vitro tests are not fully predictive of in vivo safety and efficacy [32]. In vivo testing remains an important step to evaluate pathological bone and tissue response. Different species vary in bone metabolism and remodeling [33]. Large animal species, which are widely used in the orthopedics field are dogs, sheep, goats, and pigs [33,34]. Although the composition and mass of bones in pigs and dogs mimic human bone to some extent, they both have drawbacks when used in orthopedic research. For pigs, high bone growth rates, excessive body weight, and behavioral issues lead to a high incidence of bone abnormalities [34,35]. For dogs, some studies have mentioned disadvantages such as a significantly higher bone mineral density (BMD) (BMD value in dog bone: ~340 mg/cm^3^ vs. BMD value in human bone: ~180 mg/cm^3^), variability in bone turnover, and ethical concerns [34,35]. In the present study, sheep were used. Table 2 summarizes the positives and negativities of sheep utilization for orthopedic purposes.

Mehrvar et al.’s [43] study is the first published investigation on the in vivo response of sheep bone to Al-free GPC formulated from the patented glass by Towler et al. (US 7,981,972) ((wt.%): SiO_2_: 41.79, ZnO: 42.45, CaO: 9.75, SrO: 6.00) [26]. The results of sheep bone response to this material were promising, suggesting that the formulated Al-free GPC has the potential to be used as a new bone void filler.

To further investigate the in vivo response of different GPC compositions, in this study, two tantalum-containing Al-free GPCs of Glass A (wt.%): SiO_2_: 23.79, ZnO: 32.32, CaO: 4.63, SrO: 16.73, P_2_O_5_: 8.99 and Ta_2_O_5_:13.50) (GPCA) and Glass B (wt.%): SiO_2_: 38.75, ZnO: 38.81, CaO: 4.52, SrO: 11.14, P_2_O_5_: 3.81 and Ta_2_O_5_: 2.97) (GPCB) were designed. It must be noted that the glass composition used by Mehrvar et al. was naive of Ta_2_O_5_ and P_2_O_5_ compounds [43]. Moreover, the powder:liquid (P:L) ratio of the fabricated GPC was different (glass: 3 g, acid: 0.9 g, water: 0.9 mL) compared to GPCA and GPCB (glass: 10 g, acid: 7.5 g, water: 7.5 mL). Both GPCA and GPCB were formulated based on the patented, tantalum-containing glass proposed by Towler and Alhalawani (US 10,815,144) [30]. Alhalawani et al. reported a significant reduction in sternal instability for this composition following several rheological, mechanical, and in vitro tests [44]. GPCA was formulated with a higher amount of incorporated Ta (compared to GPCB) due to promising in vitro results reported for biomaterials containing tantalum [45,46,47]. GPCB formulation was previously used as a control GPC in Hasandoost et al.’s study [48].

In the present study, the rheological, compressive strength, ion release, pH, and cytotoxicity of GPCA and GPCB were measured and analyzed. According to the results of these tests, one GPC composition was selected for the sheep trial and the in vivo response to the implanted material was investigated. For further analysis, the results of the present study were compared with the GPC used by Mehrvar et al. [43].

## 2. Materials and Methods

### 2.1. Glass and Cement Preparation

The glasses used for preparation of both GPCA and GPCB were supplied by a glass manufacturer (Mo-Sci, Rolla, MO, USA) using specific compositions (Table 3) covered by the patent by Towler and Alhalawani (US 10,815,144) [30]. The GPCs were prepared by mixing poly (acrylic acid) (PAA, Mw: ~35,000, median particle size <90 µm, Advanced Healthcare Ltd., Tonbridge, UK) with specific amounts of the glass powder (Glass A or Glass B) and DI water (Table 3). The P:L ratio was 10:15. Moreover, Table 3 shows the composition of Glass C, along with the GPCC formulation and ratio used in Mehrvar et al. [43].

### 2.2. Working and Setting Times

The working and setting times of GPCA and GPCB were measured in ambient air (23 ± 1 °C) according to ISO 9917-1:2007 for dental-based cements [49]. The working time was measured, using a stopwatch, from the start of mixing the powder and liquid until the GPC was in the malleable dough stage ready for use.

To measure the setting time, a 10 mm × 8 mm × 5 mm mold was set on aluminum foil and filled with the mixed GPCs. After 60 s of mixing, the assembly was placed on a metal block (8 mm × 75 mm ×100 mm) in an oven at 37 °C. Then, 90 s after mixing, a 400 g needle indenter was pressed on the surface of the GPC (25 °C) for 5 s and then removed. This process was repeated every 30 s until the needle was unable to make a complete indent. The net setting times of the five tests were recorded.

### 2.3. SEM-EDS Analysis

Cross-sectional images of GPCA and GPCB were captured by a JEOL Co. JSM-6380LV (JEOL Ltd., Tokyo, Japan) scanning electron microscope. An EDX Genesis Energy-Dispersive Spectrometer (JEOL Co. JSM-6380LV, JEOL Ltd., Tokyo, Japan) was used to analyze the GPC compositions.

### 2.4. Ion Release and PH

GPCA and GPCB samples (6 mm high, 2 mm diameter) (*n* = 5) were prepared according to Section 2.1 and incubated in 10 mL of DI water at 37 °C. To conduct the ion release test, five sets of each GPCA and GPCB samples were immersed in 10 mL of water for 24 h to obtain Day 1 values. The samples were then discarded. To obtain the 7-day data, five different sets of GPCA and GPCB samples were immersed in 10 mL of water for seven days and then discarded. A final set of GPCA and GPCB samples were immersed in 10 mL of water for 30 days; the samples were discarded at the end of the experiment. The ion release test was done in DI water and not in the physiological solution as GPC contact with physiological ion concentrations could affect the degradation of the glass, and the glass ions will react with the physiological solution. pH changes in the GPCA and GPCB solutions were measured using a pH meter (Corning life sciences, Acton, MA, USA) (*n* = 5). The pH meter was calibrated by employing two pH buffer solutions upon the test: 4.00 ± 0.02, 7.00 ± 0.02 (Fisher Scientific, Pittsburgh, PA, USA). The ion release rate of the GPCA and GPCB specimens was measured using inductively coupled plasma optical emission spectroscopy (Optima 7300 DV ICP-OES, Perkin Elmer, Waltham, MA, USA). Calibration standards (0.5, 1, 2.5, 5, and 10 ppm) were made for every ion and DI water was used as the control.

### 2.5. Compressive Strength

The compressive strengths ($\sigma_{c}$) of 6 mm high, 2 mm diameter cylindrical samples of GPCA and GPCB (*n* = 5), were evaluated based on ISO 9917-1:2007 [49]. Each GPCA and GPCB sample was prepared as described in Section 2.1 and incubated in DI water for 1, 7, and 30 days. A United Universal Tester (STM-50KN, United Testing Systems, Inc., Huntington Beach, CA, USA) attached to a 5 kN load cell was used for conducting the compressive strength tests, using a crosshead speed of 1 mm/min. The compressive strength, *C* (GPa), can be found from the formula below,

(1) $$C=\frac{4\;\rho }{\pi \;d^{2}\;}$$

In the above formula, *ρ* is the maximum applied load (kN) at failure (rupture as the result of crack propagation) and *d* represents the sample diameter (mm).

### 2.6. Cytotoxicity Analysis

The GPC discs (12 mm diameter, 1 mm thickness, *n* = 3) were immersed in DI water for 1, 7, 14, and 30 days prior to the test. The cytotoxicity of each sample was evaluated using pre-osteoblast cells after 24 h in culture. The GPCs were sterilized by immersing in 70% ethanol for 24 h, after UV light exposure for 2 h on each side. MC3T3-E1 preosteoblast mouse cells (ATCC CRL-2593, Oakville, ON, CA) were maintained in alpha-MEM media (Gibco) supplemented with 10% fetal bovine serum (FBS), 100 U/mL penicillin, 100 μg/mL streptomycin, and 0.25 μg/mL amphotericin (5% CO_2_, 100% humidity, 37 °C) until 80% confluent. The cement discs were placed in a 24-well plate and 50,000 cells were seeded on top. Cells were allowed to attach for 1 h in standard conditions, which was followed by the addition of 1 mL cell culture media. For the control, cells were seeded into plates (24-well) without any cement discs. Proliferation was evaluated after 24 h using the Methyl Thiazolyl Tetrazolium (MTT) Kit (11465007001, Roche Diagnostics, Mannheim, Germany) according to the manufacturer’s instructions. MTT reagent was added to each well (10% *v*/*v*) and re-incubated for 4 h. Solubilizing solution (1 mL) was added after the incubation period to dissolve formazan crystals. The absorbances of the GPCs were measured at 570 nm and 650 nm for reference with the ELISA-plate reader.

### 2.7. Sheep Preparation and Material Implantation

The animal procedure was approved by the institutional animal care committee (University of Guelph, Guelph, ON, Canada) and performed according to the protocols of the National Council on Animal Care. All procedures were performed using aseptic techniques under general anesthesia. Three Texel cross sheep (~70.6 kg) were used for this pilot study. All animals were acclimatized before surgery for seven days. Intravenous diazepam (0.3 mg/kg, Telegent, Toronto, ON, Canada) and ketamine (5.0 mg/kg, Zoetis, Kirkland, Quebec, Canada) were used for the induction of anesthesia followed by endotracheal intubation with isoflurane in a semi-closed circuit system. Following aseptic skin preparation and application of an iodine-impregnated adhesive barrier, the limbs were draped in a sterile fashion. An incision was made through the skin and subcutaneous tissue was separated using blunt dissection down to the metaphyseal bone in each leg so as to avoid adjacent neurovascular structures. A 3.5 mm pilot hole was drilled into the right femur or right tibia to a depth of 20 mm. The diameter of the hole was then increased using a 6.5 mm drill bit. Saline irrigation was used throughout the procedure to remove drilling particles and minimize the risk of infection.

After comparing the handling, compressive strength, ion release, and cytotoxicity results of each GPC formulation, three sets of GPC samples (sample 1, sample 2, and sample 3) were prepared for implantation. Prior to placing the samples in the sheep, all GPC components (glass, water and acid) and PMMA were sterilized by gamma irradiation at the dosage of 25 kGY (G.C. 220, 3.6 kGy/h, University of Toronto, Toronto, ON, Canada). The components were then mixed using a spatula until the cement was completely homogeneous. PMMA bone cement (control) (Palacos Bone Cement, Heraeus Medican, Hanau, Germany) was also prepared according to the manufacturer’s specification for comparison purposes. Following injection of the cement formulations into the tibia and femur, all adverse effects (mild joint effusion and mild lameness (grade 1 out of 5)) were resolved within 24 h. Butorphanol was prescribed intra-operatively and postoperatively as needed. Two sheep were sacrificed at 12 weeks and one sheep was sacrificed at six weeks post-surgery for terminal assessments.

### 2.8. CT/micro CT Image Acquisition

GPC implants were imaged using clinical (imaging was done on live sheep) resolution computed tomography (CT) (Discovery RT, GE Healthcare, Mississauga, ON, Canada) at Day 0 and also after six- and 12 weeks post-implantation. Moreover, higher resolution micro-CT images (General Electric Medical Systems Locus Explore Scanner, Mississauga, ON, Canada) were obtained at a 45-micron isotropic pixel resolution (Kv = 80, mA = 450). Next, the image data acquired at different angles were reconstructed into a 3D model and analyzed by MicroView (Parallax Innovations, version 2.5.1, Ilderton, ON, Canada).

Bone resorption leads to lysis of cells and appears as radiolucent lines on radiographs [50]. Therefore, to detect any sign of bone resorption, clinical CT and micro-CT images were investigated in order to check the existence of radiolucent lines at the implant–bone interface.

### 2.9. Statistical Analysis

One-way analysis of variance (ANOVA) followed by Tukey’s post-hoc tests were employed to analyze the mean differences of the gathered data using Minitab 17 (Minitab Inc., State College, PA, USA). Statistical significance was assumed if *p* values were <0.05.

## 3. Results

### 3.1. Working and Setting Times

**Figure 1:**
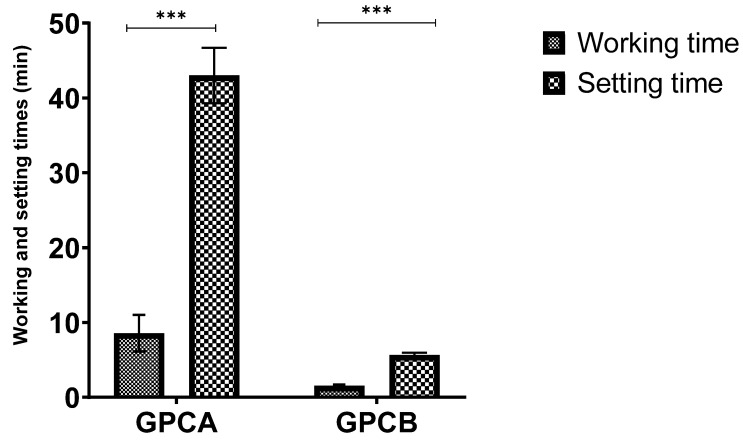
Working and setting times for GPCA and GPCB. Error bars represent the standard deviation (*n* = 5). Stars demonstrate statistical significance between the samples (*** *p* < 0.001).

Figure 1 shows the working (t_w_) and setting times (t_s_) of GPCA and GPCB. Both the working and setting times of GPCB were considerably shorter than GPCA (*p* = 0.0001).

### 3.2. SEM-EDS Results

**Figure 2:**
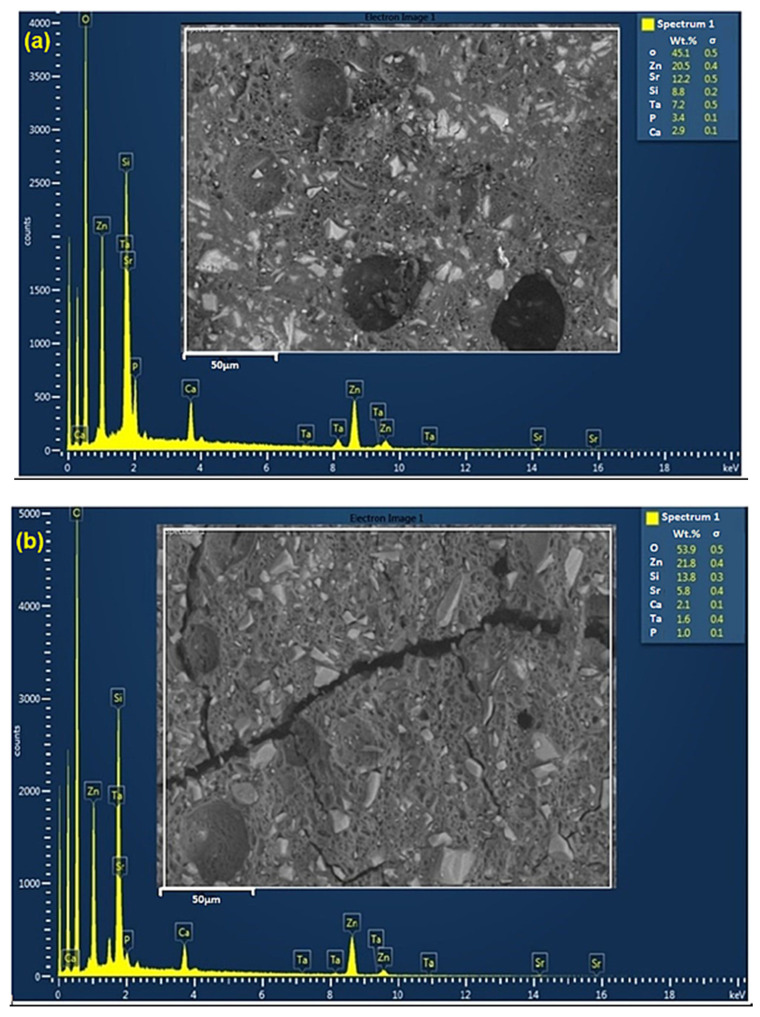
Backscattered SEM-EDS images from cross-section of GPCA (**a**) and GPCB (**b**). The white square in the SEM images indicates the interfacial areas that were used to identify the corresponding EDS spectra and the chemical composition (*n* = 3) of the GPC. Quantitative elemental composition (wt.%) can be found on the top right of the images. The images show that both GPCA and GPCB contain Zn, Si, Ca, Sr, Ta, and P elements. GPCA has a higher amount of Ta compared to GPCB.

Cross-sectional SEM-EDS analysis was used to provide the quantitative elemental analysis (wt.%) of the GPCA and GPCB series after 30 days of immersion in DI water (Figure 2). SEM revealed the existence of pores (≤10 μm), and small bright particles embedded in the GPC matrix. These bright spots are unreacted glass particles encircled by a cross-linked matrix that act as reinforcement and transfer the load within the matrix. In the maturation process, the diffusion of glass cations into the carboxylic acid continues for months, which leads to the improvement in the mechanical properties of the cement. Moreover, as the GPC matures, the ratio of non-evaporable to evaporable water increases and the dehydration and excessive humidity in GPC decrease as it matures, which improves the GPC mechanical properties [13,51]. Micro-cracks are apparent for the image obtained from the GPCB composition. Both GPCA and GPCB contain Zn, Si, Ca, Sr, Ta, and P. The most significant wt.% differences were related to Ta, Sr, and Si. GPCA contained a higher amount of Ta (7.2 wt.%) compared to GPCB (1.6 wt.%). Moreover, the amount of Si in GPCA was less than GPCB (8.8 wt.% for GPCA vs. 13.8 wt.% for GPCB). Another significant difference was related to the Sr content, which decreased more than 6 wt.% in GPCB.

### 3.3. PH Values

**Figure 3:**
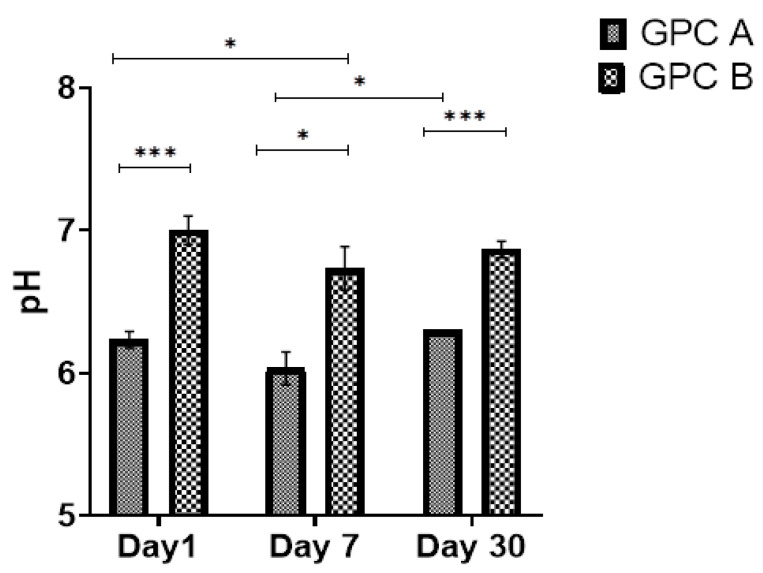
pH measurements of GPCA and GPCB solutions after maturation in DI water for 1,7 and 30 days, Error bars represent standard deviation from the mean (*n* = 5). Stars demonstrate statistical significance between the samples (* *p* < 0.05, *** *p* < 0.001).

The changes in pH for GPCA and GPCB after 30 days of incubation can be found in Figure 3. There were significant differences between GPCA and GPCB on Day 1 (*p* = 0.0001), Day 7 (*p* = 0.003), and Day 30 (*p* = 0.0001). No statistically significant difference was observed for GPCB from Day 1 to Day 30. Moreover, statistically significant differences were observed between Day 1 vs. Day 7 (*p* = 0.038) and Day 7 vs. Day 30 (*p* = 0.011) for GPCA.

### 3.4. Ion Release Profiles

**Figure 4:**
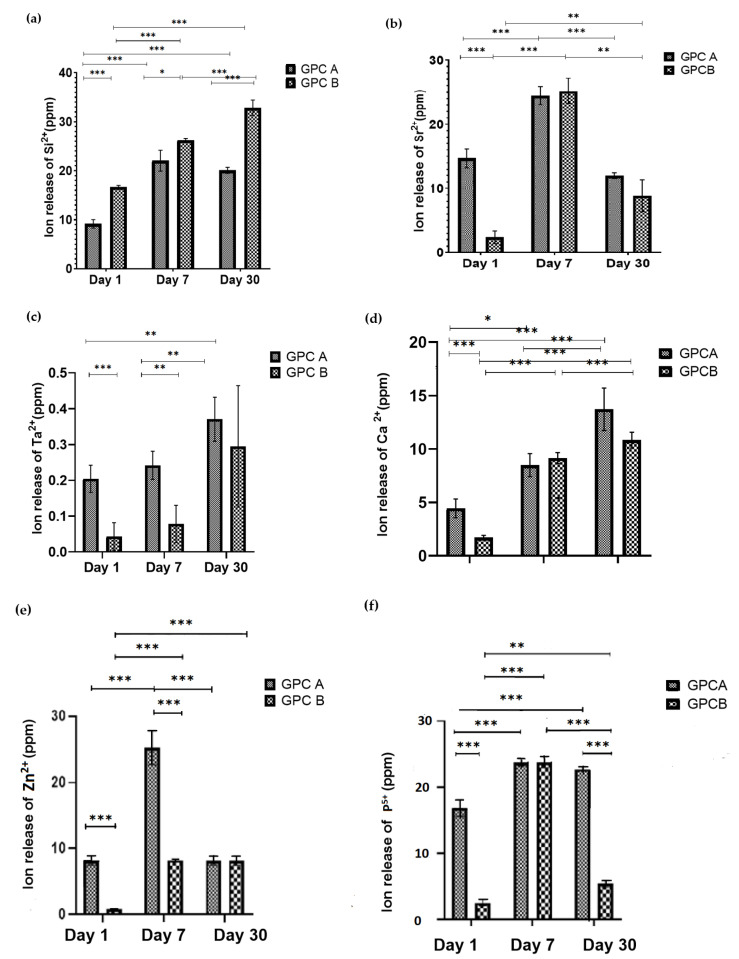
Ion release profiles of GPCA and GPCB after incubation in DI water for 1, 7 and 30 days. Error bars represent standard deviation (*n* = 5). Stars demonstrate statistical significance between the samples (* *p* < 0.05, ** *p* < 0.01. *** *p* < 0.001). (**a**) Ion release of Si^2+^ after 1, 7, and 30 days incubation in DI water (**b**) Ion release of Sr^2+^ after 1, 7, and 30 days incubation in DI water (**c**) Ion release of Ta^2+^ after 1, 7, and 30 days incubation in DI water (**d**) Ion release of Ca^2+^ after 1, 7, and 30 days incubation in DI water (**e**) Ion release of Zn^2+^ after 1, 7, and 30 days incubation in DI water (**f**) Ion release of P^5+^ after 1, 7, and 30 days incubation in DI water.

Figure 4 represents the ion release rate of Si^2+^, Ta^2+,^ Ca^2+^, Sr^2+^, Zn^2+^, and P^5+^ after 1, 7, and 30 days incubation in DI water for GPCA and GPCB. As can be seen, the highest ion releases on Day 1 from both GPCA and GPCB were pertinent to P^5+^ (16.91 ppm from GPCA) and Si^2+^ (16.68 ppm from GPCB), respectively. Moreover, from Days 1 to 30, Si^2+^ showed the highest release from GPCB (32.85 ppm). The releases of Ca^2+^ and Ta^2+^ demonstrated an increasing trend for both GPCs. On Day 1, the release of Ca^2+^ from GPCA was more than that from GPCB (*p* = 0.006) due to the higher Ca content in GPCA compared to GPCB. The release of Ca^2+^ for GPCA showed a steady increase between Day 1 and Day 30 (*p* = 0.009) and Day 7 to Day 30 (*p* = 0.029). A similar upward trend was observed between Day 1 and Day 30 (*p* = 0.0001) and Day 7 to Day 30 (*p* = 0.009) for GPCB. The overall ion release of Ta^2+^ for both GPCA and GPCB was negligible. As expected, Ta^5+^ release for GPCA was considerably more than GPCB (*p* = 0.007). This trend continued for Day 7 (*p* = 0.01). Nevertheless, no significant differences were observed between both groups on Day 30. GPCA and GPB were significantly different on Day 1 and Day 7 (*p* = 0.0001) with respect to Zn^2+^ release. Moreover, GPCA demonstrated significant differences at Day 1 vs. Day 7 and Day 7 vs. Day 30, while GPCB was different at Day 1 vs. Day 7, and Day 1 vs. Day 30 (*p* = 0.0001).

### 3.5. Compressive Strength

**Figure 5:**
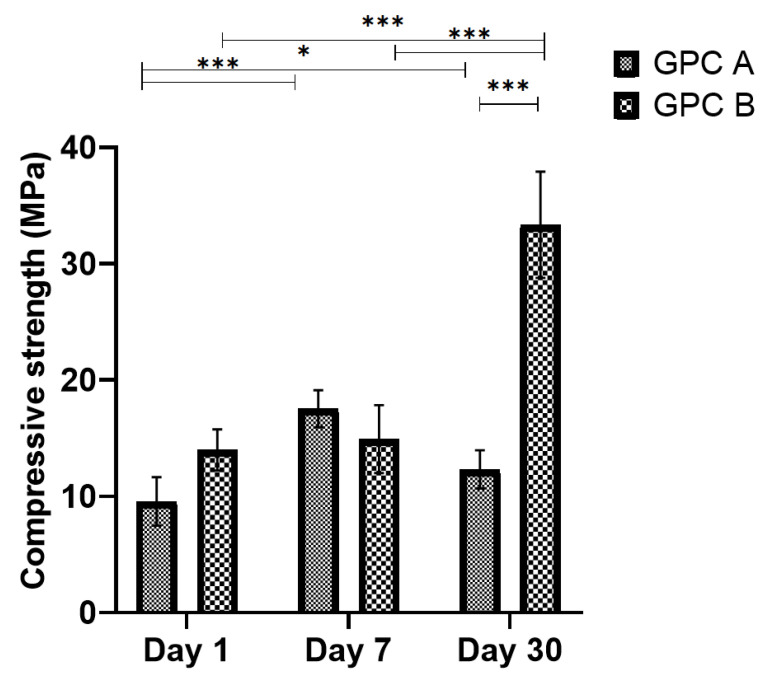
Compressive strengths of GPCA and GPCB after maturation in DI water for 1, 7, and 30 days. Error bars represent standard deviation (*n* = 5). Stars demonstrate statistical significance between the samples (* *p* < 0.05, *** *p* < 0.001).

The compressive strengths (σ_c_) of the two GPC series after 1, 7, and 30 days incubation in DI water are shown in Figure 5. Compressive strength was influenced by maturation time for both GPCA and GPCB. The only significant difference between GPCA and GPCB in compressive strength was observed at Day 30 (*p* = 0.0001), being significantly higher (33.3 MPa) for GPCB than GPCA (12.3 MPa).

### 3.6. Cytotoxicity

**Figure 6:**
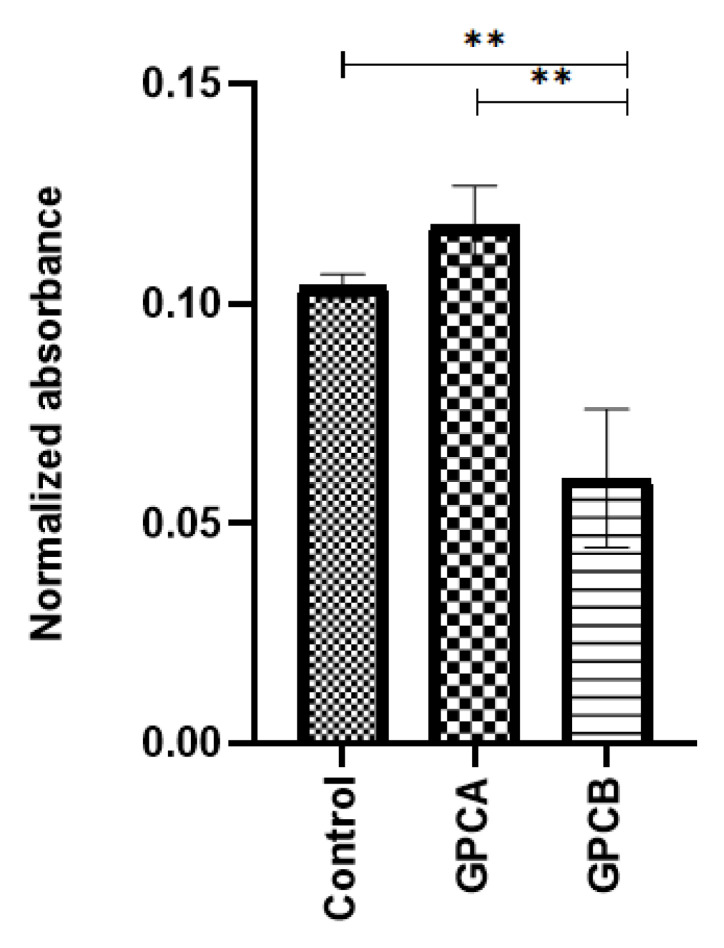
MTT assay results of pre-osteoblast cells in contact with GPCA and GPCB disk surface against control after 24 h of culture. Error bars represent standard deviation (*n* = 5). Stars demonstrate statistical significance between the samples (** *p* < 0.01).

Figure 6 shows the cell viability results for each sample tested after 24 h of culture. The results were compared to control (preosteoblast cells and media) No change in cell numbers was observed for GPCA compared to the control, but significant loss of cells can be seen for GPCB (*p* = 0.001). Moreover, there was a significant difference between the absorbance values of the control vs. GPCB (*p =* 0.006).

**Figure 7:**
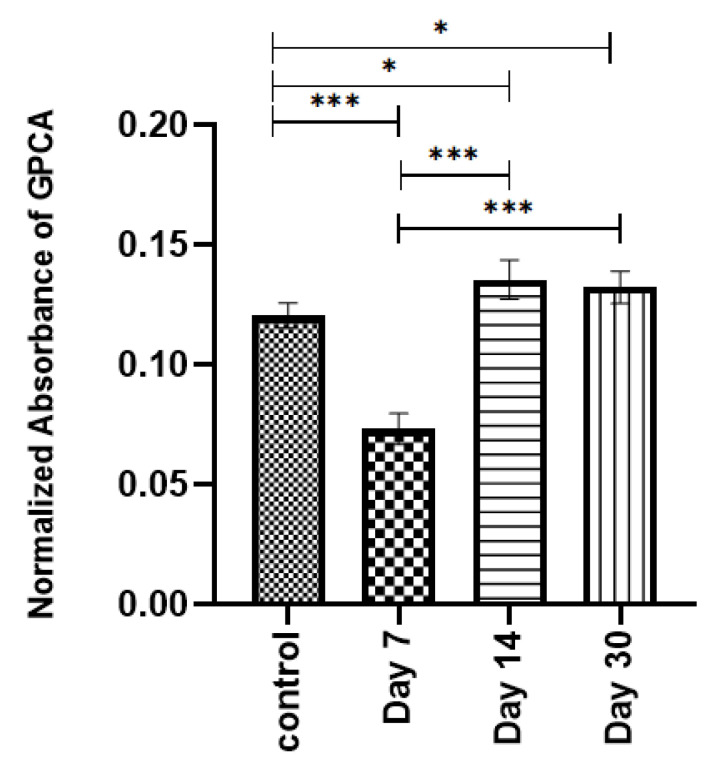
MTT assay results of pre-osteoblast cells in contact with GPCA disk surface against control (preosteoblast cells and media). Error bars represent standard deviation (*n* = 5). Stars demonstrate statistical significance between the samples (* *p* < 0.05, *** *p* < 0.001).

Furthermore, the cytotoxicity test was extended for GPCA formulation until Day 30 and compared with the control (media and preosteoblast cells). Figure 7 represents the cell viability results for GPCA. The cells were cultured for 24 h on samples pretreated by immersion in DI water for 7, 14, and 30 days. A significant drop in MTT activity compared to the control sample was observed at Day 7. However, after 14 and 30 days, cells showed significantly higher metabolic activity than that of the control.

Overall, GPCA showed a more acceptable working time, setting time and ion release (e.g., Ta^2+^, Ca^2+^, Sr^2+^) compared to GPCB. Additionally, the MTT assay showed no change in pre-osteoblast cell numbers for GPCA (compared to control) after 24 h, while a reduction of cells was seen for GPCB. Therefore, these characteristics made GPCA a more attractive choice over GPCB for the in vivo testing.

### 3.7. Bone Response to Implanted GPCs Using CT and Micro-CT Scan Analysis

**Figure 8:**
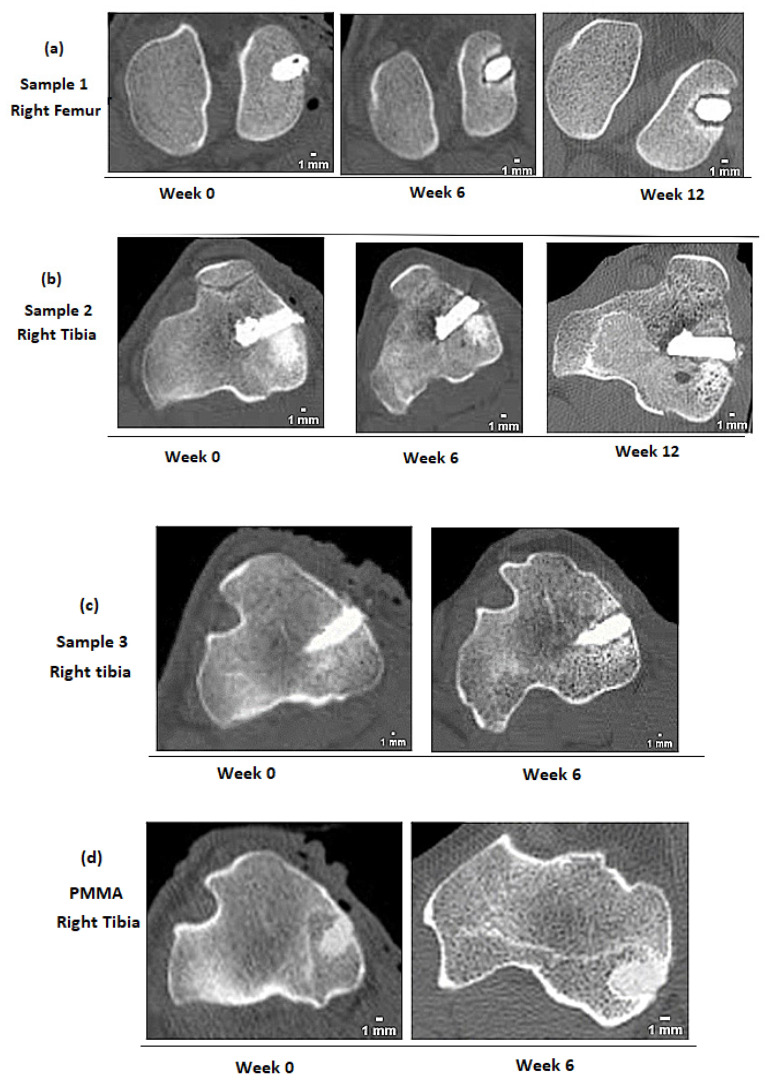
Clinical CT images of GPCA and PMMA samples implanted in the sheep bone at different time intervals. The images show varied response of bone to implanted GPCA. The radiopacity of PMMA appears less than GPCA. (**a**) Clinical CT mage of Sample 1 (right femur) at 0, 6 and 12 weeks (**b**) Clinical CT image of Sample 2 (right tibia) at 0, 6 and 12 weeks (**c**) Clinical CT mage of Sample 3 (right tibia) at 0, and 6 weeks (**d**) Clinical CT mage of PMMA (right tibia) at 0 and 6 weeks.

**Figure 9:**
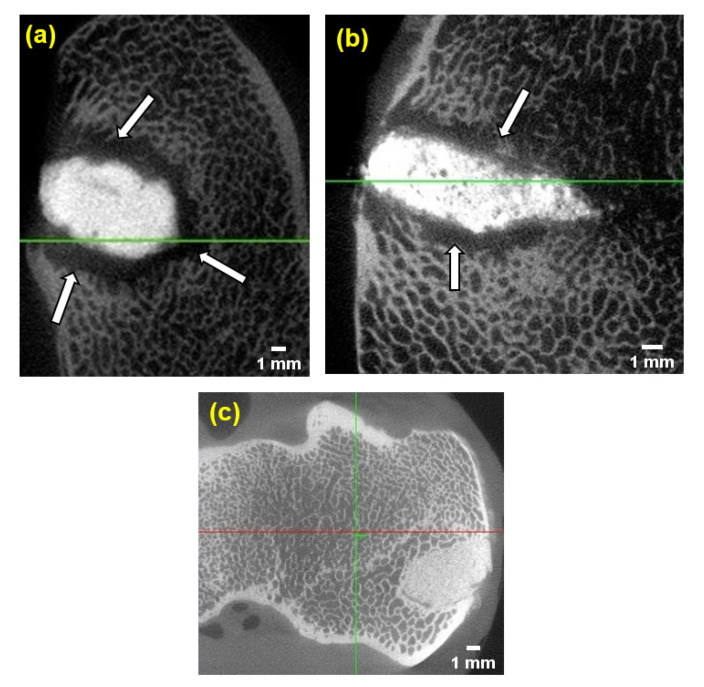
(**a**) shows a micro-CT image of GPCA sample 1 at 12 weeks, while (**b**,**c**) show micro-CT images of sample 3 and PMMA at six weeks post-implantation, respectively. Extensive bone resorption at the implant site can be seen for sample 1 (indicated by arrows), while sample 2 showed minor resorption. The radiopacity of both GPCA samples was higher than that of PMMA.

Figure 8 and Figure 9 show clinical and micro-CT images of implanted GPCA and PMMA over time. For sample 1, at the time of implantation, a homogeneous mass of GPC can be seen (Figure 8a). After six weeks, resorption between GPCA and bone caused radiolucent zones surrounding the entire component. The severity of this resorption appears to increase from week 6 to week 12. However, sheep bone showed a different response to samples 2 and 3. In sample 2, mild bone resorption can be seen surrounding the implant at week 6 (Figure 8b). At week 12, a continuous bone–cement interface along the implant without any evidence of resorption is visible, indicating an acceptable radiological response. This suggests that any resorption seen at week 6 is accounted for by further bone deposition and remodeling by week 12. Moreover, sample 3 showed a slight lucent border surrounding the implant, indicating minimal bone resorption occurred from week 0 to week 6 (Figure 8c).

The micro-CT images of GPCA and PMMA are presented in Figure 9. For this analysis, the sheep were sacrificed at the end of six weeks (sample 3 and PMMA) and 12 weeks (sample 1).

Two images were taken after the sheep were sacrificed at six weeks and one image was obtained after 12 weeks post-implantation. As can be seen in Figure 9a,b, both GPCA samples were visually more radiopaque than PMMA (Figure 9c). PMMA is not a radiopaque material, therefore radiopacifiers such as barium sulphate (BaSO_4_) or zirconium dioxide (ZrO_2_) are usually added in order to make the cement radiopaque [52]. These radiopacifiers are not part of the polymeric chain, whereas in GPC, ions such as Sr^2+^ exist in the glass phase, which makes the GPC more radiopaque compared to PMMA [53].

The lucent border (indicated by arrows) around sample 1 identifies severe resorption. However, only a slight lucent border surrounding sample 3 (similar to Figure 8c) was observed, which indicates that a mild degree of resorption occurred. A continuous bone–cement interface with no sign of resorption was observed around the defect filled with PMMA (Figure 8d) and (Figure 9c).

## 4. Discussion

PMMA is used for anchoring implants to bone, for fracture fixation, and for management of non-critical bone defects (≤5 mm) [6]. GPCs may be able to overcome some of the limitations (e.g., thermal necrosis, volumetric shrinkage) that currently exist with using PMMA in rTKA. For example, while PMMA acts as a grout in vivo and only provides mechanical interlock between the bone and implant [6], GPCs chemically bind to the mineral phase of bone through the ion exchange. Al-free GPCs have been developed by the authors and set by an acid-base reaction between calcium–zinc silicate (CaO–ZnO–SiO_2_) glass, PAA, and water. Glass plays an important role in the setting of GPC. According to the glass compositions (Table 3), all glasses contained SiO, ZnO, CaO, and SrO. However, the glass used by Mehrvar et al. (glass C) did not contain P_2_O_5_ and Ta_2_O_5_ [43].

The random network theory proposed by Zachariasen is widely used to describe the glass structure and formation based on the observation of oxides [54,55]. Zachariasen considered oxides such as P_2_O_5_ and SiO_2_ as network formers that form the bulk of a glass structure [56]. Moreover, according to this theory, network modifiers such as CaO and SrO alter the covalently bonded glass network [56]. Several studies have proven that SrO can be replaced by CaO as both Ca^2+^ and Sr^2+^ have similar ionic radii and play the same chemical role in the glass network [57,58,59]. Moreover, some oxides can play intermediary roles (e.g., ZnO, Ta_2_O_5_) and function as both network formers and modifiers. Therefore, the glass composition is an important factor, which determines the properties of GPCs.

In the present study, GPCA contains a considerably more Ta compared to GPCB, which was confirmed by SEM/EDS analysis (Figure 2). The oxygen ions in transition metals are closely packed with metal ions located in tetrahedral or octahedral holes within the oxygen ions. Ta^5+^ ions enter the silicate network and form isolated six-fold coordination, TaO_6_ [60,61]. By increasing Ta^5+^ content (glass A), TaO_6_ octahedra share corners and form higher Ta–Ta links (compared to glass B). This leads to longer working times and setting times for GPCA as there would be a delay in cross-linking of the COOH groups and Ta^5+^. Moreover, GPC B contains more Si (i.e., more bridging oxygen, which leads to an increase in cross-linking between COO^–^ groups and metal cations), leading to shorter working and setting times. The glass particle size in GPCB (10–20 µm) was less than in GPCA (30–45 µm), meaning that GPCB had a higher surface area for reaction and subsequently a shorter setting time.

Based on the pH data in Figure 3, it can be concluded that GPCB showed significantly higher pH (compared to GPCA samples) after 30 days of maturation in DI water. The acidity of the solution depends on the rate of ions leaching from the surface of the GPC. It can be assumed that the incorporation of Ta_2_O_5_ in the precursor glass leads to a rapid release of unstable residual glass particles (Ta has a highly reactive surface). Therefore, the higher content of Ta in GPCA is one of the factors leading to a prolonged period of lower pH.

According to Figure 5, GPCB compressive strength showed an increasing trend from Days 1 to 30. The strengthening mechanism of GPCs is based on the cross-linking of carboxyl groups (COOH) in the polymeric acid to the ions released from the glass surface [62]. This process is continuous and leads to an increase in GPC strength over time [63]. The lower compressive strength of GPCA (compared to GPCB) after 30 days is related to the incorporation of more Ta into GPCA than GPCB. As mentioned earlier, there would be a delay in cross-linking of COOH groups and Ta^5+^. Thus, a higher amount of Ta leads to lower compressive strength in GPCA compared to GPCB.

Figure 6 shows a better performance of GPCA in terms of cytotoxicity response compared to GPCB. Several studies have reported that Ta^2+^ ions stimulate cell proliferation [64,65,66]. In a study conducted by Andreson et al., atomic force microscopy (AFM) was used to investigate cell volumes (volumes of the substrate-attached cells) in pre-osteoblast cells attached to a Ta substrate. The results confirmed an increase of 50% in pre-osteoblast cell volume, which was attached to the planar Ta substrate [64]. Additionally, Ta was found to increase cell adhesion and proliferation compared to titanium (Ti) surfaces [65] due to the higher surface energy of Ta (Ta = 55 mNm^−1^ vs. Ti = 42 mNm^−1^) [67]. Apart from Ta, the other ions in GPCA such as Ca^2+^, Sr^2+^, P^5+^, and Zn^2+^ showed higher release rates compared to GPCB. The release of these therapeutic ions is beneficial. For example, Ca^2+^ ions increase bone mineral growth and are a potential regulator in wound healing [68,69]. P^5+^ ions act as a network former and also play an important role in the formation of hydroxyapatite [70]. Ito et al. reported that the incorporation of 12% mol Zn to a composite of tricalcium phosphate (TCP) and hydroxyapatite (HA) considerably promoted osteoblastic cell proliferation and alkaline phosphatase activity of rat cells [27]. It is not known which ion had more impact on increasing the viability of GPCA compared to GPCB. More investigation is required to study the impact of releasing each ion on the chemical structure and cytotoxicity of GPC.

The Mehrvar et al. study is the first published investigation on the in vivo response of sheep bone to Al-free GPCs [43]. Therefore, in this study, the results of bone response to GPCA were compared to those from Mehrvar et al. [43]. Comparing the handling properties and compressive strength in both studies showed that GPCA and Mehrvar’s GPC composition (namely GPCC, Table 3), apart from different compositions and ratio, had similar setting times (~ 34–43 min) and initial compressive strengths (~ 10–14 MPa). CT and Micro-CT analysis of implanted GPCC used in the Mehrvar et al. study, (at 8 and 16 weeks, respectively) showed a homogeneously mixed cement with a very small lucent border around the implant. Moreover, the implanted materials increased the bone mineral density. Mehrvar et al. [43] attributed this increase in bone mineral density to the release of Sr^2+^ ions. It has been proved that the appropriate dosage of Sr can increase bioactivity, bone healing and osteointegration [28,71]. However, the excess release of Sr^2+^ ions is found to be toxic to the bone and is reported to disturb calcium metabolism [72]. Moreover, the GPCC composition was formulated using a higher molecular weight PAA (M_w_ = 210 k) compared to GPCA (M_w_ = 50 k). The increase in the molecular weight of PAA intensifies matrix strength as the length of the polymer chain will be increased. Moreover, the P:L ratio of GPCC is higher than that of GPCA which causes an increase in the GPC cohesive strength.

For further analysis of the sheep response to GPCA, the maximum toxicity levels of Zn^2+^, Si^2+^, Sr^2+^, P^5+^, Ca^2+^, and Ta^2+^ ions reported in the literature were compared to the maximum ion release of GPCA and GPCB (Table 4).

As can be seen in Table 4, most ion concentrations measured in the present study were far from toxic levels. However, the Zn^2+^ toxicity level reported in the literature seems to be very close to the maximum concentration of Zn^2+^ in GPCA. Studies have indicated that the addition of low levels of Zn in biomaterials enhances cell proliferation and protein synthesis [27,79,80]. Brauer et al. showed that the addition of Zn has a dose-dependent effect on the cytotoxicity of GPC, on which the addition of 300 μM of Zn increased mouse osteoblast metabolic activity, while the GPC exposed to 400 μM of Zn showed a reduction in cell metabolic activity [73]. Therefore, excessive Zn^2+^ ion release can be considered as a factor in causing bone resorption. As can be seen in Figure 4, Zn^2+^ release was maximized (~25 ppm) after GPCA was immersed in water for seven days. The number of pre-osteoblastic cells was also reduced significantly at Day 7 (Figure 7). Therefore, the decrease in pre-osteoblast cell numbers is speculated to be derived from the increased toxicity of Zn^2+^. Another cause of resorption might be related to the low pH reported for GPCA. Low pH is proven to stimulate osteoclast activities [81]. The acidity level depends on the ions leaching from the surface of GPCA, therefore, the composition of cement plays an important role in improving the response of bone to these materials. Different GPCA samples (samples 1, 2, and 3) triggered varied reactions to the bone, which might be related to mixing variability and difficulty in drilling and inserting the material into different parts of the sheep bone. Although sheep models are well-accepted for orthopedic studies, sheep do not precisely represent human anatomy and have different bone turnover rates. The structures of bone in sheep and humans are different [34]. In terms of bone density, sheep bones have significantly higher density compared to human bones (almost 1.5–2 times greater than that of human bone). Therefore, the implanted materials could have different results if tested on human bones.

## 5. Conclusions

In this paper, the handling properties, compressive strength, ion release, pH, and cytotoxicity of two GPCs (GPCA and GPCB) formulated from the SiO_2_-CaO-SrO-P_2_O_5_-Ta_2_O_5_ glass system were evaluated. The GPCA formulation with a higher concentration of Ta showed more working time (8.5 ± 2 min) and setting time (43 ± 3 min). The longer working and setting time provides additional time for surgery. Additionally, the release of ions (e.g., Ta^2+,^ Ca^2+^, Sr^2+^, P^5+^) from the GPCA in the first day was greater than GPCB. This has a positive effect on accelerating wound healing as well as bone growth. Additionally, the MTT assay showed no change in pre-osteoblast cell numbers for GPCA (compared to control) after 24 h, while a reduction of cells was seen for GPCB. Therefore, GPCA was selected for in vivo testing. Clinical CT and micro-CT images of three implanted GPCA versus PMMA at 0, 6, and 12 weeks after surgery revealed the varied response of sheep bone to GPCA. One of the three GPCA samples did not show any resorption at 12 weeks post-implantation with a continuous bone–cement interface (Figure 8b). However, one was associated with mild bone resorption only around the implant site compared to PMMA after six weeks (Figure 8c) and the third sample showed extensive resorption (Figure 8a). High levels of Zn^2+^ release, low pH, and difficulty in inserting the samples into different parts of the sheep bone are speculative factors that might be involved in the varied response observed for implanted GPCA samples.

In this study, the maximum concentration of ions was compared to the maximum concentration of ions reported in the literature. However, the ion release test was based on the number of ions in 10 mL of water, which is different than the concentration of ions in vivo.

The results presented in this study are based on preliminary pilot experiments conducted to determine the in vivo response of sheep bone to the implanted material. Further in vitro studies are required to modify GPC formulations with the focus on reducing the rate of Zn^2+^ release and pH. Following modification of GPC, toxicity assays should be performed to evaluate each GPCA ion’s toxicity level in vitro. Moreover, more information about the degradation and toxicity of the implanted materials can be obtained through the blood analysis. Finally, the biocompatibility of the formulated GPC should be evaluated using small animal models prior to further sheep surgery.

## Figures and Tables

**Table 1:** Description, characteristics, and treatment options of different bone fillers currently available in rTKA [6].

Description	Bone Defect Depth	Treatment Option
Minor and contained cancellous bony defects	<5 mm depth	Poly(methyl methacrylate) (PMMA) fill, morselized allograft or autograft
Defects in one femoral condyle or one tibial plateau	5–10 mm depth	Morselized allograft or metal augments
	10–20 mm depth	Metal augments, metaphyseal sleeves, structural allografts
Both femoral condyles or tibial plateaus are damaged	<20 mm depth	Metal augments, metaphyseal sleeves, structural allografts, custom-made prostheses, cones
Deficient metaphyseal segment; a bone loss that comprises a major portion of the condyle or plateau	>20 mm depth	Structural allografts, custom-made component, cones

**Table 2:** Advantages and disadvantages of sheep utilization for in vivo orthopedic research.

Advantages	Disadvantages
Realistic mechanical loads acting on the limbs [36]	Higher maintenance and handling costs compared to certain animal models (e.g., rabbits) [37]
Similar body weight, and metabolism to humans [32,36]	Denser trabecular bone and different microstructure compared to humans (BMD values for human bone: ~180 mg/cm^3^ vs. BMD value for sheep: ~440 mg/cm^3^) [32,38] Slower bone turnover compared to human bone
Easily available, ethically better accepted than dogs [39]	-
Tested successfully as load-bearing bone defect models (femur, tibia, ulna) [37,39,40]	-
Suitable for evaluation of potential treatments for osteoporosis [41]	-
Comparable tibial blood supply (young sheep) [42]	-

**Table 3:** Glass, cement compositions, and formulations.

Glass Formulations	SiO_2_	ZnO	CaO	SrO	P_2_O_5_	Ta_2_O_5_	Cement Formulation [Glass (g):PAA (g):DI Water]
Glass A (wt.%)	23.79	32.32	4.63	16.73	8.99	13.50	10 g:7.5 g:7.5 mL PAA molecular weight: 50 k Glass particle size: 30–45 µm
Glass B (wt.%) [48]	38.75	38.81	4.52	11.14	3.81	2.97	10 g:7.5 g:7.5 mL PAA molecular weight: 50 k Glass particle size: 10–20 µm
Glass C (wt.%) [43]	41.79	42.45	9.75	6.00	-	-	3 g:0.9 g:0.9 mL PAA molecular weight: 210 k Glass particle size: 45–63 µm

**Table 4:** Toxicity level of ions presented reported in the literature versus maximum concentration of ions in GPCA and GPCB.

Ion	Toxicity Level (Reported in the Literature)	Effect	Maximum Concentration of Ions in the Present Study (GPCA and GPCB)
Zinc (Zn)	˃26.1 PPM [73]	Cell death [73]	25.2 PPM
Silicate (Si)	˃224.6 PPM [74]	Inhibits the nucleation of hydroxyapatite [74]	32.8 PPM
Strontium (Sr)	˃700.9 PPM [75]	Decreases bone mineralization [75]	25.2 PPM
Phosphorous (P)	˃41 PPM [76]	Cell death [76]	23.8 PPM
Calcium (Ca)	˃235 PPM [77]	Decreases matrix mineralization [77]	13.7 PPM
Tantalum (Ta)	˃25 PPM [78]	Decrease in cell viability [78]	0.4 PPM

## Data Availability

The data presented in this study are available on request from the corresponding author.
